# Clinical characteristics and in silico analysis of congenital pseudarthrosis of the tibia combined with neurofibromatosis type 1 caused by a novel *NF1* mutation

**DOI:** 10.3389/fgene.2022.991314

**Published:** 2022-09-28

**Authors:** Jingfang Xu, Ying Zhang, Kun Zhu, Jiabin Li, Yuelin Guan, Xinyu He, Xuejing Jin, Guannan Bai, Lidan Hu

**Affiliations:** ^1^ Department of Orthopaedics, The Children’s Hospital, Zhejiang University School of Medicine, National Clinical Research Center for Child Health, Hangzhou, China; ^2^ Institute of Translational Medicine, Zhejiang University School of Medicine, Hangzhou, China; ^3^ Department of Pathology, The Children’s Hospital, Zhejiang University School of Medicine, National Clinical Research Center for Child Health, Hangzhou, China; ^4^ Department of Pharmacy, The Children’s Hospital, Zhejiang University School of Medicine, National Clinical Research Center for Child Health, Hangzhou, China; ^5^ The Children’s Hospital, Zhejiang University School of Medicine, National Clinical Research Center for Child Health, Hangzhou, China; ^6^ Centre for Evidence-based Chinese Medicine, Beijing University of Chinese Medicine, Beijing, China; ^7^ Department of Child Health Care, The Children’s Hospital, Zhejiang University School of Medicine, National Clinical Research Center for Child Health, Hangzhou, China

**Keywords:** neurofibromatosis type 1, congenital pseudarthrosis of the tibia, ferroptosis, bone deformity, genetic mutation

## Abstract

Congenital pseudarthrosis of the tibia (CPT) is a rare congenital bone malformation, which has a strong relationship with Neurofibromatosis type 1 (NF1). NF1 is an autosomal dominant disease leading to multisystem disorders. Here, we presented the genotypic and phenotypic characteristics of one unique case of a five-generation Chinese family. The proband was CPT accompanied with NF1 due to *NF1* mutation. The proband developed severe early-onset CPT combined with NF1 after birth. Appearance photos and X-ray images of the left limb of the proband showed significant bone malformation. Slit-lamp examination showed Lisch nodules in both eyes of the proband. Whole-exome sequencing (WES) and Sanger sequencing confirmed the truncation variant of *NF1* (c.871G>T, p. E291^*^). Sequence conservative and evolutionary conservation analysis indicated that the novel mutation (p.E291^*^) was highly conserved. The truncated mutation led to the loss of functional domains, including CSRD, GRD, TBD, SEC14-PH, CTD, and NLS. It may explain why the mutation led to a severe clinical feature. Our report expands the genotypic spectrum of NF1 mutations and the phenotypic spectrum of CPT combined with NF1.

## Introduction

Congenital pseudarthrosis of the tibia (CPT) is a rare type of bone deformity with typical characteristics of pseudarthrosis in early life, pathological fractures of the anterolateral part of the tibia that result in bowing, narrowing of the medullary canal, or a cyst (Crawford, 1986; Hefti et al., 2000). The prevalence is approximately one in 140,000–250,000 births (Crawford and Schorry, 1999; Kesireddy et al., 2018). Previous experience and research suggested that there was a strong relationship between CPT and neurofibromatosis type 1 (NF1) (Crawford, 1986), but the prevalence was heterogeneous worldwide (Hefti et al., 2000; Vander Have et al., 2008). A recent review by van Royen et al. (2016) reported that the prevalence of NF1 in patients with CPT was around 84.0%.

NF1 is caused by mutations in the *NF1* tumor suppressor gene on the 17q11.2 chromosome and affects multiple systems including neurocutaneous and skeletal systems characterized by various clinical manifestations including the typical Café-au-lait macules, Lisch nodules, bone deformity, and multiple neurofibromas (Young et al., 2002; Jett and Friedman, 2010). Treatment for CPT is very difficult and challenging, which is often referred to as one of the most puzzling and frustrating conditions in pediatric orthopedics worldwide (Granchi et al., 2012). Patients may need multiple surgeries frequently to consolidate the pseudarthrosis and unfortunately, refractures occur. After the surgery, there is still a risk of not achieving bone union, and the risk of amputation will be never completely avoided. CPT complicated with NF1 makes the treatment even more challenging, which significantly impairs patients’ growth and development, mental health, and quality of life, which imposes a heavy burden on children, families, and society. The etiology of CPT has not been completely understood and little is known about why CPT is closely related to NF1. Hereditary factors play an essential role in pathogenesis.

The tumor suppressor gene *NF1* comprises 350 kb of genomic DNA (Viskochil et al., 1990) and contains 60 exons encoding a large neurofibromin protein of 2,818 amino acids (Marchuk et al., 1991). To date, pathogenic mutations in the *NF1* gene have been detected as point mutations, frame deletions or duplications, indels, and complex rearrangements (Human Gene Mutation Database (HGMD, http://www.hgmd.org/). Most of these variations result in the truncated protein product. Thus, the NF1 deficiency or the loss of neurofibromin function is related to the increased Ras activity and a high proliferation rate (Trovó-Marqui and Tajara, 2006). The development of Whole-exome sequencing (WES) makes the diagnosis and potential targeting of gene therapy possible. Here, we reported a novel truncated variant (c.871G>T; p. E291^*^) in a Chinese pedigree and elucidated the possible functional loss through bioinformatic analysis.

## Materials and methods

### Proband, pedigree, and clinical assessments

This study was conducted according to the principles of the Declaration of World Medical Association (2013). It has been approved by the Local Research Ethics Committee of the Children’s Hospital, Zhejiang University School of Medicine (2022-IRB-148). In March 2022, a boy of 14 years and 11 months visited the Department of Orthopedics in the Children’s Hospital, Zhejiang University School of Medicine, Hangzhou, China. The main complaints were deformities of the left ankle and the left limb after birth and the inability to walk due to the severe pain in the left limb for 2 weeks. He was considered as the proband in the present study. In addition, he had scoliosis of the spine and multiple café-au-lait macules all over the body except for the face. Comprehensive examinations and tests were conducted, including X-ray examinations of limbs and spine, three-dimensional computed tomography (CT) of the left tibia and fibula, visual examinations and slit-lamp examination, blood routine examination, liver function, renal function, routine urine test, routine stool test, bone metabolism test, Vitamin D test, blood clotting function test, and detections for Human Immunodeficiency Virus (HIV), Syphilis, Hepatitis B, and Hepatitis C. The family history of NF1 and CPT was asked by the orthopedist and information on the five-generation Chinese family was collected. Based on the above information, the diagnosis of the proband was made as CPT combined with NF1. The proband underwent tibial osteotomy and correction and intramedullary nail fixation. At present, a small amount of callus growth can be seen. His mother and grandmother (i.e., his mother’s mother) were observed with café-au-lait macules, dermatofibroma, freckles, and severe scoliosis and kyphosis (only seen in the mother) and a diagnosis of NF1 was made according to the establishing criteria. Grandfather (i.e., mother’s father), father, and younger brother of the proband were also included in the present study. They had a relatively good health status without any CPT and NF1-related symptoms or signs.

### Whole-exome sequencing

Genomic DNA was extracted from peripheral leukocytes using the Blood Genome Qiagen Blood DNA mini kit (Qiagen). The whole-exome DNA library was prepared by using VAHTS Universal DNA Library Prep Kit for Illumina V3 (Vazyme) and KAPA HyperExome Sequence Capture Kit (Roche, United States). The Illumina DNA Standa ds and Primer Premix Kit (kapa) was used for library quantification, followed by sequencing with the dnBSEQ-T7 gene sequencer (PE150). Raw data with low-quality reads were filtered for quality control. The Burrows-Wheeler Aligner (BWA) sequence alignment method was performed by comparing with the human genome reference (hg19). The mutation sites in the target sequence were identified using GATK software. Familial segregation analysis of identified mutations was carried out whenever applicable. The variants were validated by Sanger sequencing analysis by using TaKaRa LA PCR™ Kit Ver.2.1 (TaKaRa) with ABI 3500XL (Applied Biosystems) platform. The primers were as follows: TGT​AAA​ACG​ACG​GCC​AGT​TTG​CCC​TTG​GGT​TTT​TAC​ATA​G (forward) and CAG​GAA​ACA​GCT​ATG​ACC​CCA​TCA​AAC​AAA​GAA​ACC​TAA​AAT​GA (reverse).

### Mutation bioinformatics analysis

The American College of Medical Genetics and Genomics (ACMG) Standards and Guidelines for the interpretation of sequence variants were followed in this study (Kalia et al., 2017). The homodimer structure of neurofibromin in open conformation was downloaded from the Protein Data Bank (PDB ID: 7R04). The Pymol visualization tool (http://www.pymol.org/) was used to generate the monomer of NF1. The sequence alignments were analyzed by examining multiple sequence alignments using the program Consurf server and Unipro UGENE.

## Results

### Clinical information

**FIGURE 1:**
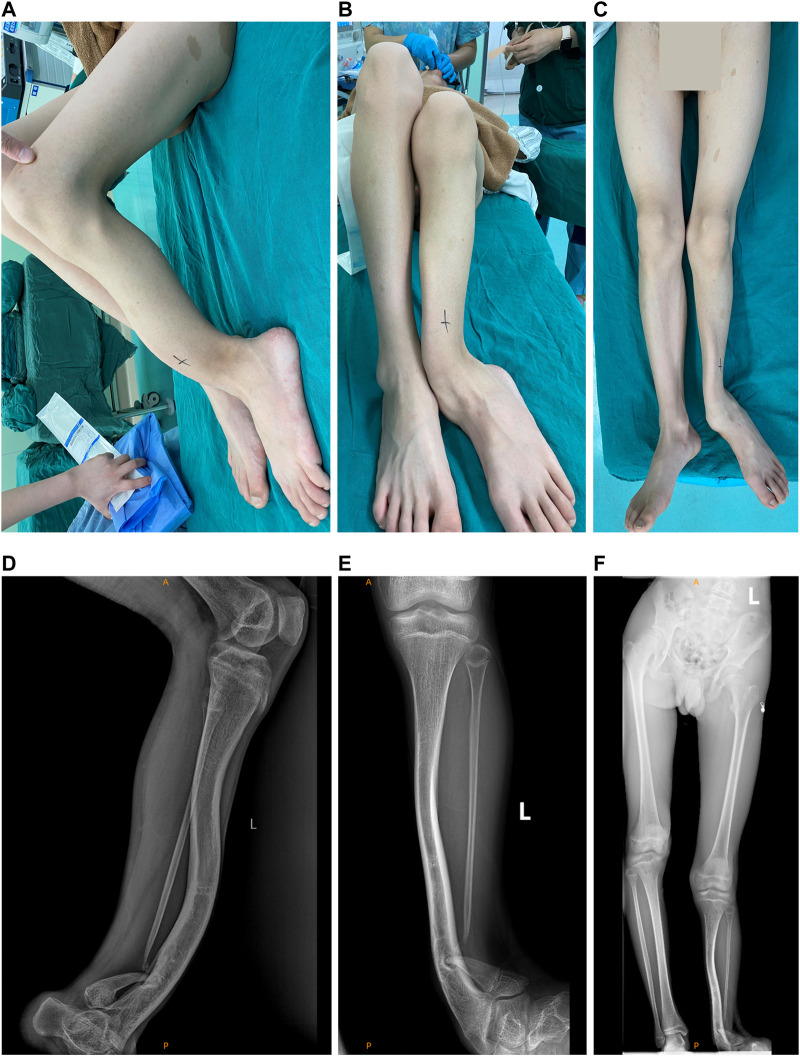
Appearance photos and X-ray images of the left limb of the proband. **(A)** Lateral photo of the pseudarthrosis of left tibia. **(B)** Photo of pseudarthrosis of the left tibia and the left ankle valgus. **(C)** Photo of the unequal lengths of the left and right limb. **(D)** X-ray image of the pseudarthrosis of left tibia. **(E)** X-ray image of the pseudarthrosis of the left tibia and the left ankle valgus. **(F)** X-ray image of the unequal lengths of the left and right limb.

**FIGURE 2:**
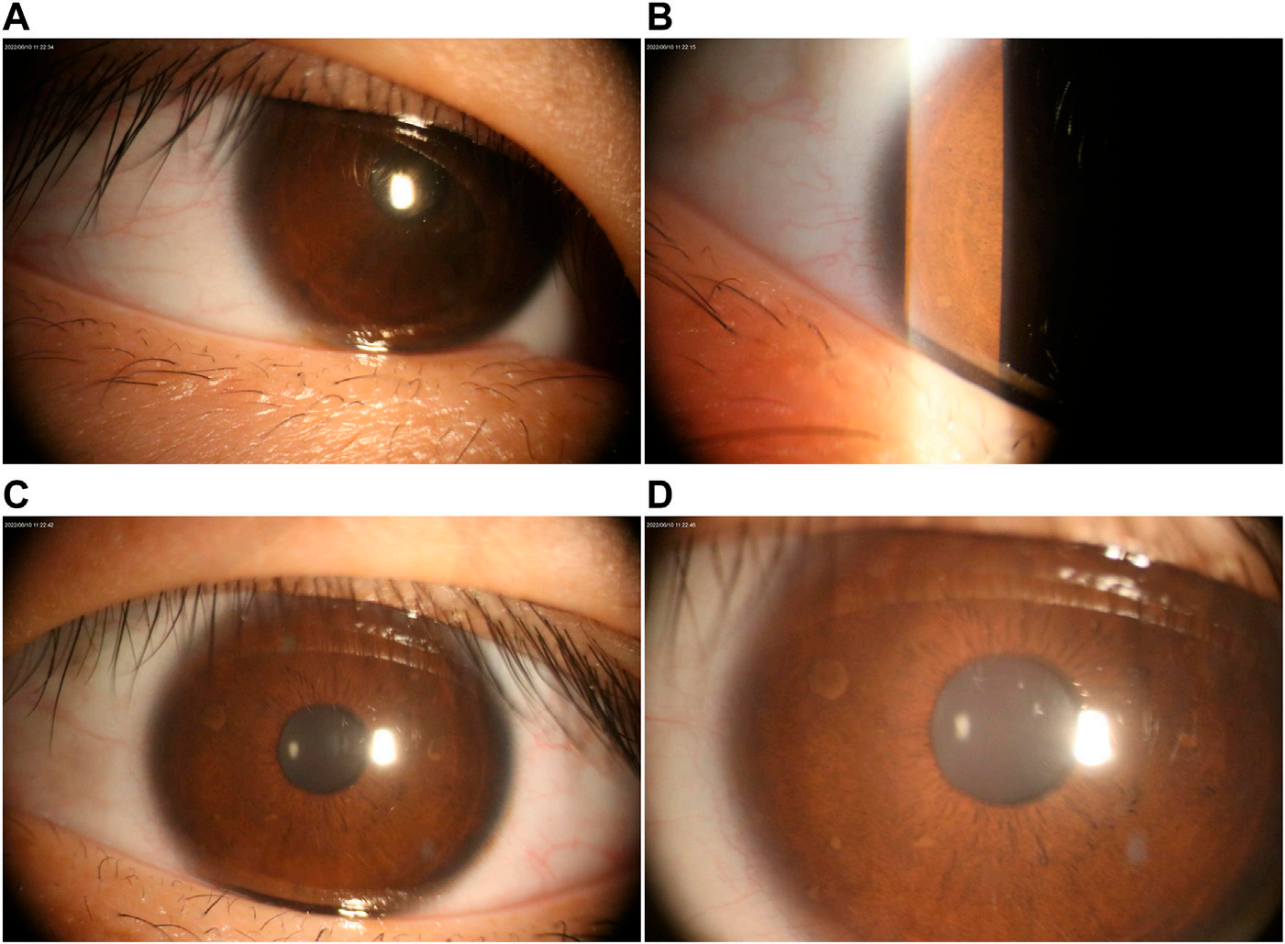
Ophthalmological examination under the slit lamp. **(A–B)** Lisch nodules in the left eye. **(C–D)** Right eye with multiple Lisch nodules.

**FIGURE 3:**
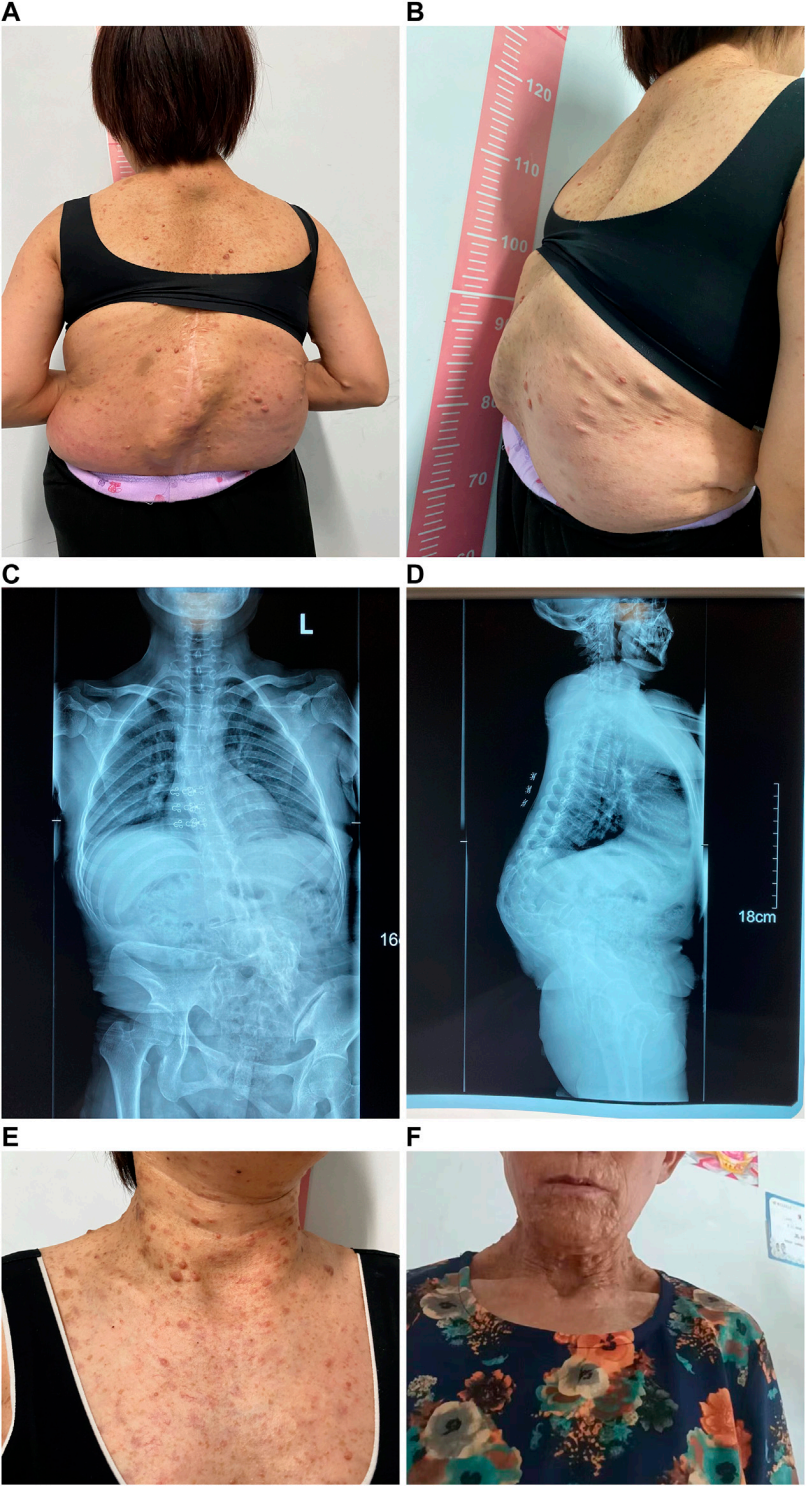
Appearance photos and X-ray images of proband’s mother. **(A)** Photo of the back with multiple café-au-lait macules, dermatofibroma, and severe scoliosis and kyphosis. **(B)** Lateral photo of the back showing severe scoliosis and kyphosis. **(C)** X-ray image of spinal scoliosis. **(D)** X-ray image of spinal scoliosis and kyphosis. **(E)** Appearance photo of café-au-lait macules and dermatofibroma on the neck and front chest of the proband’s mother. **(F)** Appearance photo of dermatofibroma around the mouth of the proband’s grandmother.

**FIGURE 4:**
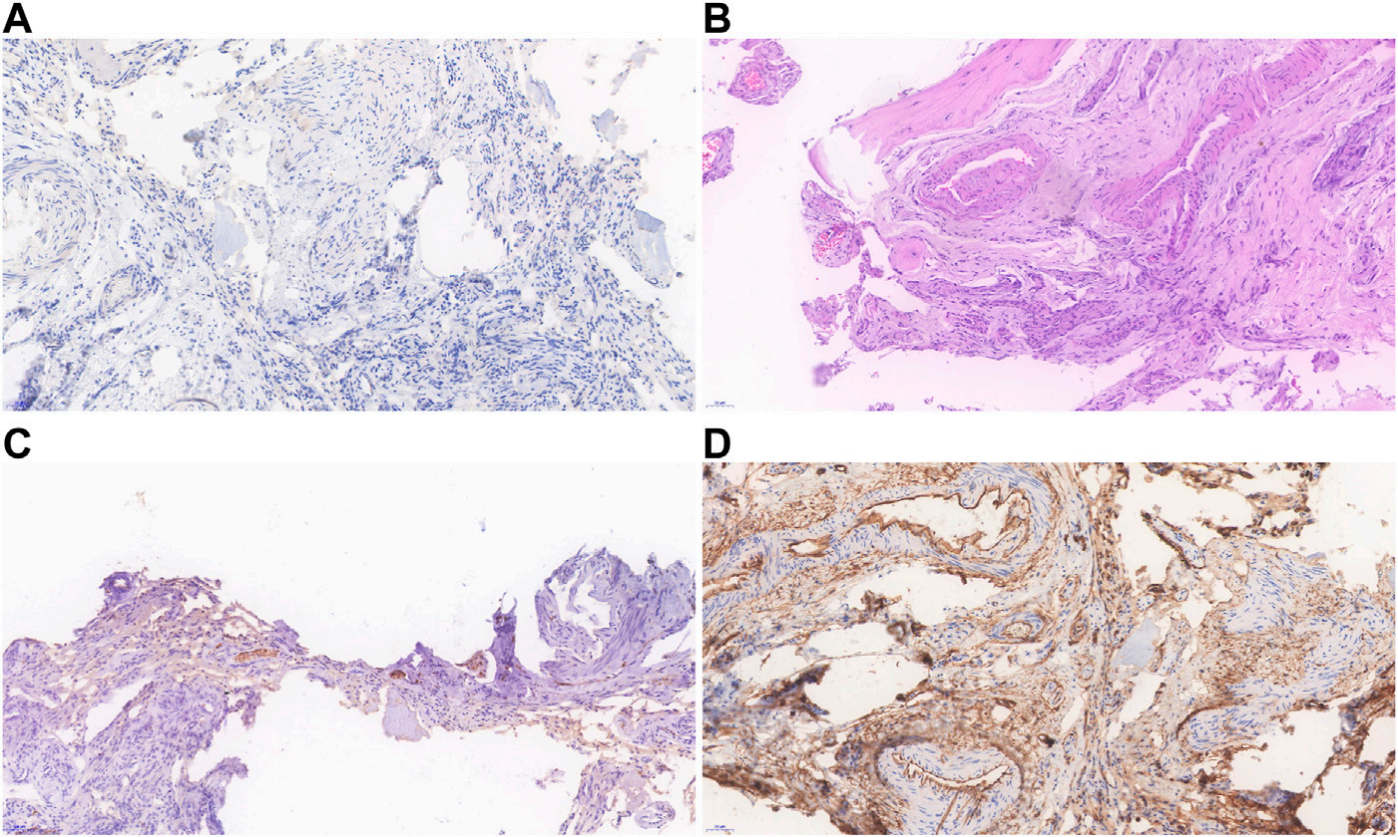
Histopathological findings of the proband’s neurofibroma. **(A)** Immunohistochemistry showed that the lymphatic marker D2-40 was negative. **(B)** Spindle tumor cells are distributed in bundles with bone destruction, and the tumor is rich in blood vessels. **(C)** Expression of S-100 was scattered in tumor. **(D)** CD34 expression was positive in vascular endothelial cells.

Table 1 presents the clinical manifestations of the three affected members. Regarding the proband (V-1), the onset age of the disease was at birth. He had bone deformities including the unequal length of lower limbs, bending of left tibia, pseudarthrosis, ankle valgus, and scoliosis of the spine. Multiple café-au-lait macules were distributed all over the body except for the face. Freckles were observed in the axilla and groin. The laboratory tests indicated a low concentration of Vitamin D in the blood and abnormal bone metabolism. The image examinations showed bone deformities, loss of bone mass, and Lisch nodules in both eyes. The mother (IV-2) and grandmother (III-4) had café-au-lait macules distributed all over the body, dermatofibroma around the mouth, on the neck, and in the front chest as well freckles in the axilla and groin. The mother of the proband had severe scoliosis and kyphosis. Figures 1–4 shows the selected results of image examinations, appearance photos, and histopathological findings. Figure 1 shows the presence of pseudarthrosis in the left tibia, valgus of the left ankle, and unequal lengths of the lower limbs. Figure 2 are photos taken under the slit-lamp showing more Lisch nodules in the right eye than that in the left eye. Figures 3A–E shows severe scoliosis and kyphosis of the proband’s mother as well as the café-au-lait macules and dermatofibroma on the neck and in the front chest. Figure 3F shows the dermatofibroma distributed around the mouth of the proband’s grandmother. Figure 4 are histopathological findings that provided supplementary information to make a diagnosis of neurofibroma.

**TABLE 1:** Basic information, clinical manifestations, laboratory tests and image tests of the three affected members.

Characteristics	V-1	IV-2	III-4
Age (years)	14.9	35	55
Onset age (years)	At birth	13	/
Sex	Male	Female	Female
Height (cm)	161	145	155
Weight (kg)	43	40	38
Clinical manifestations	1. Bone deformity	1. Café-au-lait macules	1. Café-au-lait macules
	2. Café-au-lait macules	2. Dermatofibroma	2. Dermatofibroma
	3. Axillary and inguinal freckles	3. Axillary and inguinal freckles	3. Axillary and inguinal freckles
	4. Scoliosis of spine	4. Scoliosis and kyphosis	/
Laboratory tests	1. Vitamine D: 2.91 (20–80 ng/ml)	/	
	2. β-collagen specific sequence: 2.292 (0.196–1.665 ng/ml); 25-HydroxyvitaMin D3: 38.5 (41.7–175.0 nmol/L); Bone alkaline phosphatase quality: 21.7 (23.4–119.1 ng/ml)		
Image tests	1. Pseudarthrosis, ankle valgus and foot deformity	Scoliosis and kyphosis	/
	2. Unequal length of both lower limbs		
	3. Scoliosis of spine		
	4. Loss of bone mass		
	5. Lisch nodules		

### Genetic characterization and bioinformatic analysis

**FIGURE 5:**
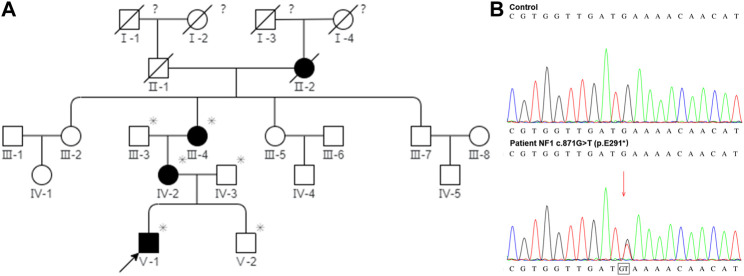
Pedigree of the CPT NF1 patient’s family and Sanger sequencing confirmed the mutation of NF1. **(A)** Segregation analysis of NF1 variants in a pedigree represents an autosomal dominant inheritance pattern. Squares and circles represent males and females, respectively. Closed and open symbols indicate affected members and unaffected subjects. The arrow denotes the proband. Family history was negative for consanguinity. The slash symbol indicates that the subject is deceased. The asterisk shows the individual underwent both clinical and genetic analyses. The question mark indicates that the ophthalmic history is not available. **(B)** Sanger sequencing at the mutation site with a blood sample. High-throughput sequencing showed a novel *de novo* truncation mutation of the *NF1* gene in the proband was identified (c.871G>T; p. E291^*^).

There are 21 members in this five-generation Chinese family and six individuals were recruited for further study, including three affected individuals and three unaffected individuals. As shown in Figure 5A, the pedigree of the CPT combined with the NF1 patient’s family was consistent with autosomal dominant inheritance. To validate the diagnosis, the proband and his brother, parents, and grandparents (i.e., mother’s parents) underwent the WES. The mean read depth reached 20x for 98.9% of the target sequences. This was further confirmed by Sanger sequencing (Figure 5B). The genetic test revealed a heterozygous truncation variant (c.871G>T, p. E291^*^) of the exon eight in the *NF1* gene (#OMIM: 162,200). The novel mutation was evaluated as a pathogenic mutation according to ACMG guidelines (2017) (Table 2).

**TABLE 2:** Variant table for the novel mutation of *NF1* gene.

Gene	Chromosome	Exon	Nucleotide change	Amino acid change	ACMG
*NF1*	Chr17:29509666	8	c.871G>T	p.E291^*^	Pathogenetic

ACMG: the american college of medical genetics and genomics.

**FIGURE 6:**
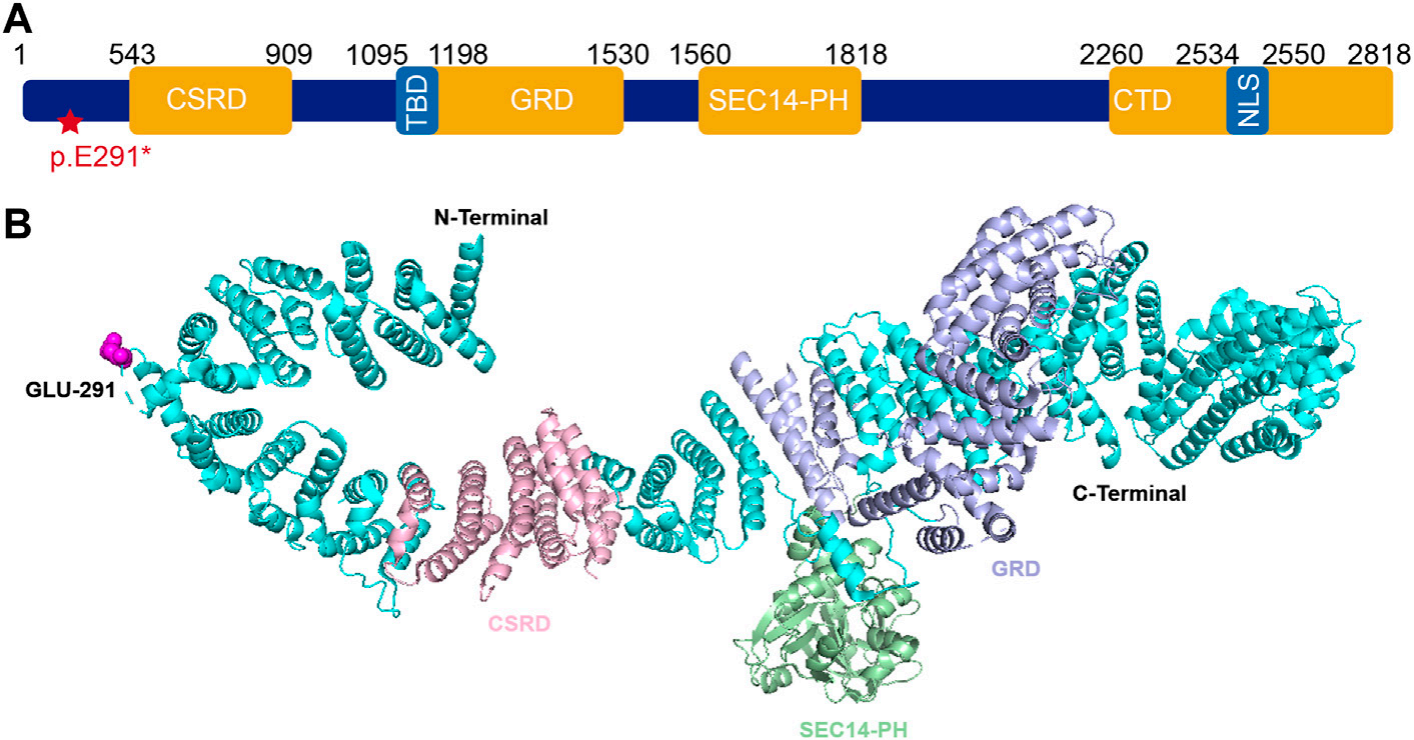
**(A)** Schematic representation of neurofibromin protein and identified mutation of *NF1* (c.871G>T; p. E291*) in this study. **(B)** The 3D structure of neurofibromin1 monomer. The NF1 was represented in cartoon and the mutation site (p.E291*) was shown as magenta sphere. The CSRD domain (residues 543–909) (light pink), GRD domain (residues 1095–1530) (light blue), SEC14-PH domain (residues 1560–1818) (pale green). Abbreviations usedare as follows CSRD, cysteine-serine-rich domain; GRD, GAP-related domain; TBD, tubulin-binding domain; SEC14-PH, Sec14-homologous domain and pleckstrin homology domain; CTD, C-terminal domain; NLS, nuclear localization signal.

**FIGURE 7:**
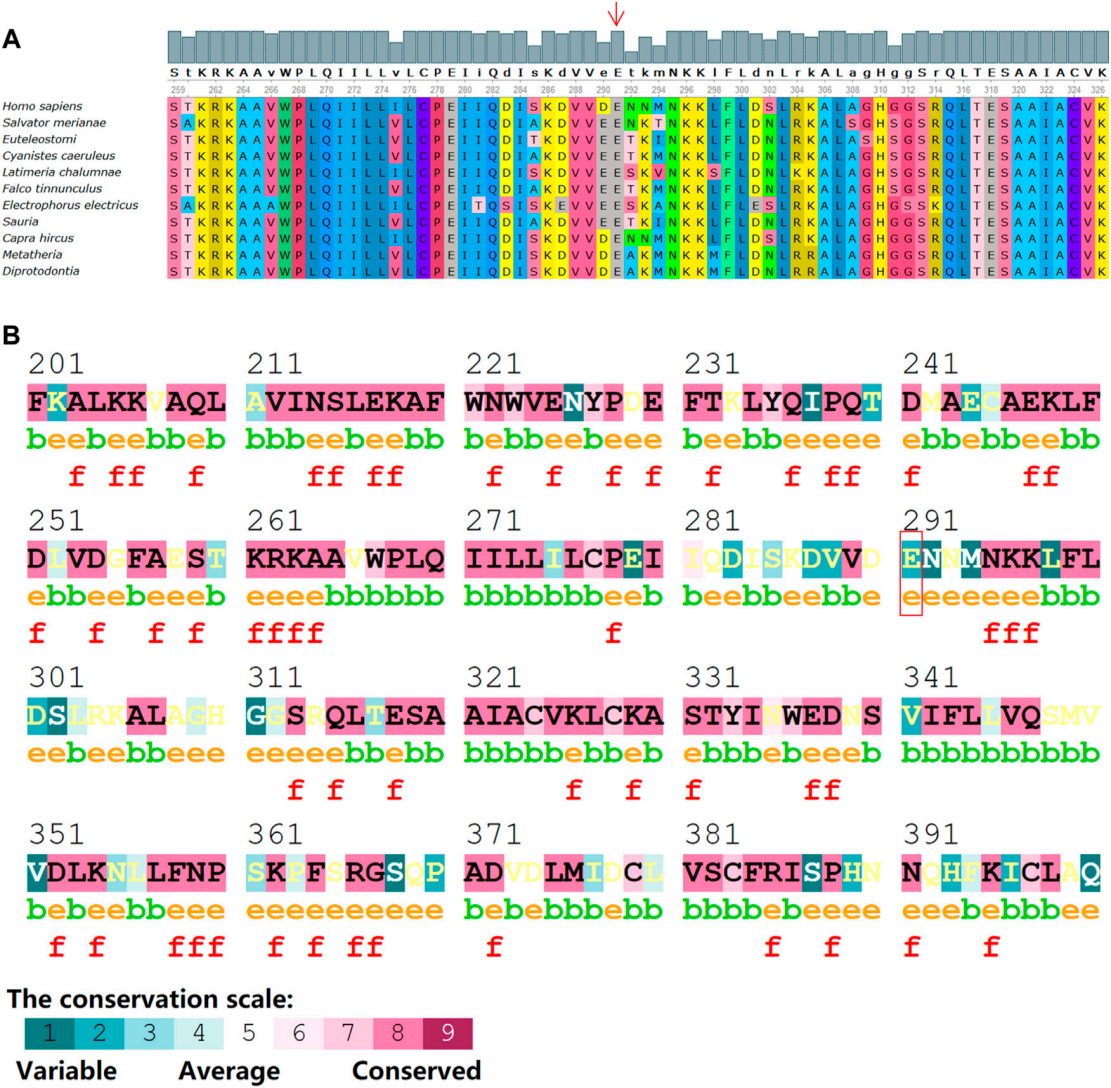
Sequence alignments of NF1 protein from various species with program Consurf server and Unipro UGENE software. **(A)** Conserved amino acids background colors are the same among species. Numbers indicate the positions of the amino acid sequences. **(B)** The mutation site (p.E291^*^) within the conservative amino acid region. The conservation scale ranges from variable (blue), to average (white) to conserved (red). Annotations: e: An exposed residue according to the neural-network algorithm. b: A buried residue according to the neural-network algorithm. f: A predicted functional residue (highly conserved and exposed).

The novel truncation mutation is segregated from the phenotype within the pedigree. This truncation mutation was located near the C-terminal of NF1 and caused the loss of most of the structure of the protein (Figure 6A). The structure of NF1 was displayed in the cartoon and the mutation site (p.E291^*^) was represented in the magenta sphere (Figure 6B). The cysteine-serine-rich domain (CSRD), GAP-related domain (GRD) and Sec14-homologous domain, and pleckstrin homology domain (SEC14-PH) were rendered light pink, light blue, and pale green, respectively. Sequence conservative analysis indicated that the highly conserved loci of the novel mutation (p.E291^*^) may be involved in essential physiological functions (Figure 7).

## Discussion

CPT is a rare disease and the epidemiological data are limited. The estimated prevalence is around one in 140,000–250,000 births. Due to the different classification systems worldwide, the prevalence or the incidence of CPT is heterogeneous across different countries. In Denmark, Andersen et al. reported a CPT incidence of 1:190,000 live births (Andersen, 1971). In Norway, the incidence of CPT was 1:60,000 when only taking into account the ethnic Norwegian patients and 1:53,000 when all patients born with CPT in Norway were included (Horn et al., 2013). A strong relationship between CPT and NF1 was suggested. A wide variation in NF1 prevalence numbers, ranging from 0.0 to 84.0%, was demonstrated by the literature review due to the various diagnosis standards (van Royen et al., 2016). Based on the NIH diagnostic criteria that have been considered the golden standard for diagnosis of NF1, the prevalence numbers of NF1 among CPT patients were reported as 42.9%, 54.7%, and 84.0% (Heikkinen et al., 1999; Hefti et al., 2000; van Royen et al., 2016). Previous studies suggested a genetic origin for the etiology of CPT combined with NF1. In this study, the symptoms of the proband were considered to be related to genetic factors because the onset age was very early, i.e., at birth, and it presented with severe symptoms. The pedigree of the CPT NF1 patient’s family indicated the pattern of autosomal dominant inheritance and Sanger sequencing confirmed the mutation of *NF1*.

CPT is one of the most puzzling pediatric orthopaedical diseases and the treatment is very challenging. Most of the patients have bending tibia within one or 2 years after birth, and they can also have fractures at birth. Tibial deformities and multiple fractures will eventually form pseudarthrosis, resulting in life-long disability. Current treatment for CPT includes conservative and surgical treatment. Before the child could walk, the physician installs a plaster bracket or a plaster tube to fix the ankle and leg. After the child could walk, the ankle and leg should be protected with lighter cast support, which may slow down the progression of CPT and avoid the formation of pseudarthrosis and the occurrence of fractures. Surgical treatment can be applied when the child gets older including pseudarthrosis tissue resection, intramedullary rod fixation, wrapped autologous iliac bone transplantation, and Ilizarov annular external fixator compression fixation. Surgery is very challenging and complicating with relatively high risks of nonunion bones, which imposes heavy burdens on the quality of life, psychological well-being, and financial situations of the patients and their families. Mladenov et al. (2020) have reported their treatment protocol for pediatric children with NF1 and tibial pseudarthrosis. Fracture union in tibial pseudarthrosis with satisfactory functional results can be achieved in more than 80% of the children (Mladenov et al., 2020).

**FIGURE 8:**
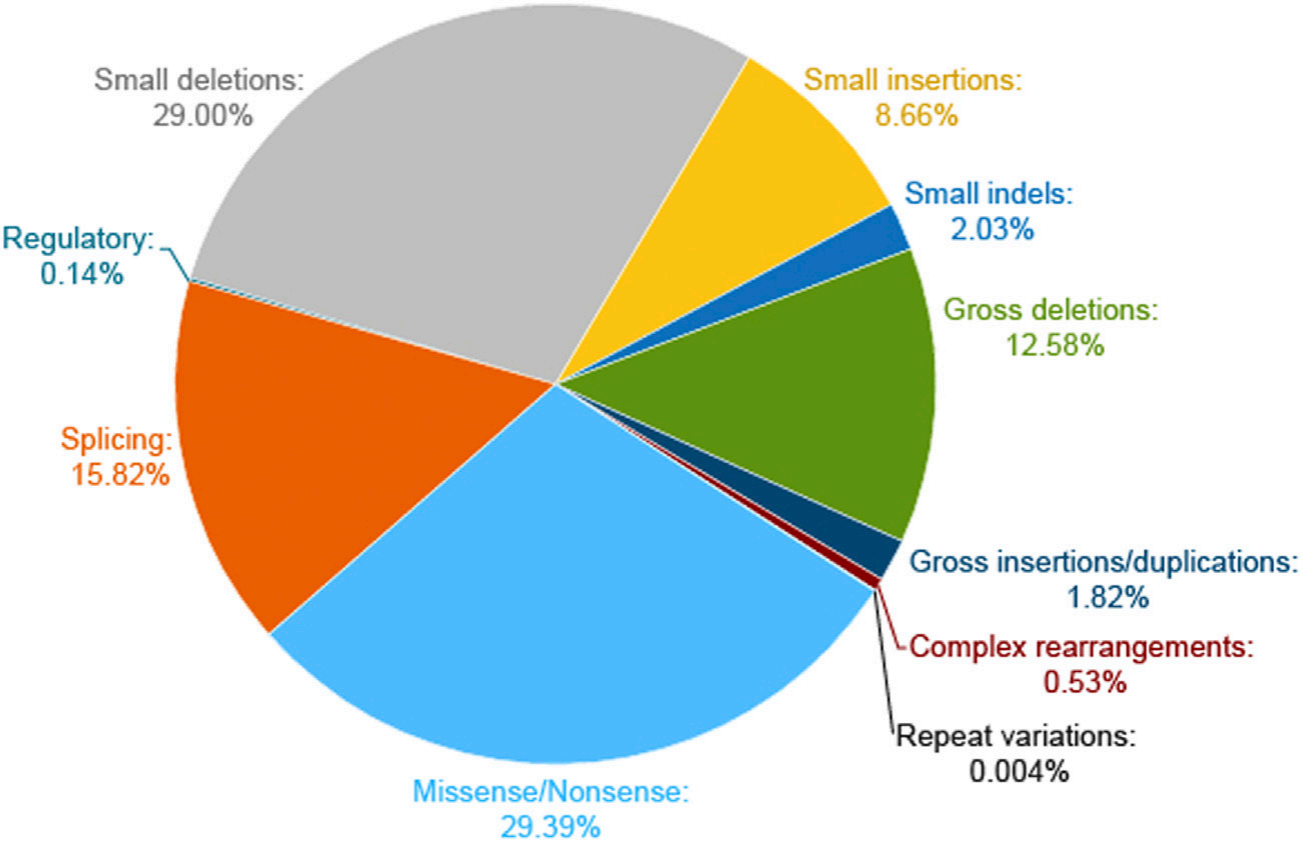
Pie chart of *NF1* mutation types on the HGMD website. The micro lesions are made up of missense/nonsense, splicing, regulatory, small deletions, small insertions, and small indels. The gross deletions consist of gross deletions, gross insertions/duplications, and complex rearrangements of repeat variations.

The WES could provide further insights into information related to the multisystem disorder. Of note, most of the reported NF1-related variants in the HGMD database are micro changes, and the majority of them are missense/nonsense and small deletions (Figure 8). Three previous studies have investigated the influence of the type of constitutional NF1 mutation on the disease phenotypic variability (Castle et al., 2003; de Luca et al., 2004; Sabbagh et al., 2013). Whereas, limited samples in the survey of Castle et al. and De Luca et al. conferred an ambiguous relationship. Sabbagh et al. revealed a fortuitous association in 565 unrelated patients from the NF-France Network. Understandably, patients with large deletions of the NF1 gene region led to a more severe phenotype. Furthermore, the tendency for truncating mutations to be associated with a greater incidence of Lisch nodules and a larger number of CAL spots as compared with missense mutations. However, these studies failed to find any statistically significant association of NF1 clinical features with mutation type. A more related survey was urgent to clarify the relationship between different genetic and clinical phenotypes.

In the present study, we found a novel truncation mutation (c.871G>T; p. E291^*^) in a pedigree with a family history of NF1 disorder. The clinical symptoms vary widely among individuals carrying *NF1* gene mutations. The highly conserved protein consists of a cysteine-serine-rich domain (CSRD), a GAP-related domain (GRD), a tubulin-binding domain (TBD), a Sec14-homologous domain, a pleckstrin homology domain (SEC14-PH) and a C-terminal domain (CTD) (Figure 6A). The pathogenic mechanism of CPT is not clear yet and the deletion or inactivation of the NF1 gene is considered as an important cause (Xu et al., 1990; Basu et al., 1992; Cichowski and Jacks, 2001; Viskochil, 2002; Brekelmans et al., 2019). NF1 gene is a tumor suppressor gene with a high mutation rate and extreme mutation heterogeneity. It encodes neurofibroma protein that enhances the GTPase activity of the RAS protein and negatively regulates the Ras/MAPK signaling pathway (Philpott et al., 2017). NF1 gene mutation causes the loss of function of neurofibroma protein, which may enhance the Ras activity, and then result in abnormal proliferation and differentiation of cell lines such as osteoclast progenitor cells and osteoblast progenitor cells (Sharma et al., 2013). The activated MAPK causes osteoblast differentiation, bone formation disorder, and the increased maturation of osteoclast cells, which also explains why fractures of CPT NF1 patients are difficult to heal (Kuorilehto et al., 2006). In other words, the fracture healing normally requires the expression of the NF1 gene that inhibits the activation of the Ras/MAPK pathway. GRD activates the activity of RasGTPase. It is well known that the multiple RAS downstream effectors including the PI3K, ERK, and RalA are highly expressed in NF1 patients. Thus, the dysfunction of GRD leads to neurofibroma tumor genesis and progression. The CSRD also regulates the GRD function *via* its phosphorylation by both kinase A (PKA) and protein kinase C (PKC) (Izawa et al., 1996; Mangoura et al., 2006). The CTD is also regulated by PKA and then regulates the Ras-GAP activity negatively (Tokuo et al., 2001; Feng et al., 2004). The functional domain SEC14-PH within the C-terminal region might be implicated in protein and lipid trafficking (Mousley et al., 2007).

The novel truncation mutation (c.871G>T; p. E291^*^) has not been reported by other literature. Besides our finding, an early study by Zhu et al. (2019) has identified 25 novel variants among 44 NF1 CPT patients in China. On exon 8, there were two novel mutations, i.e., c.731–2A > C and c.786_787insTT (p. (Lys263Leufs*19), which were interpreted as pathogenic according to the ACMG criteria (Zhu et al., 2019). Our results and previous findings have confirmed that the NF1 loss-of-function variant is a major factor leading to NF1 CPT. In the present study, we did not detect the lesion tissue. Zheng et al. (2022) collected the periosteum tissue from the pseudarthrosis site of six patients with NF1-CPT (i.e., probands) and their unaffected parents. They found that five of six NF1-CPT patients (83.3%) had NF1 inactivation in tissues. In addition, we had to admit that WES may not detect all the NF1 variants. For instance, non-coding variants from the regulating area of NF1 may be among the undetected genetic regions (Zheng et al., 2022). Therefore, we recommended using comprehensive detection and analysis of other variants using both the lesion tissue and the blood of patients with and without NF1 CPT.

The Glu^291^ is located within the N-terminal domain of neurofibromin. This truncated variant resulted in the loss of a series of domains including CSRD, GRD, SEC14-PH, and CTD. The functions of these domains might explain the clinical phenotypes in this pedigree to some extent. Although the research on the *NF1* gene has been well studied in recent years, current clinical therapies still mainly rely on symptomatic treatment and surgery, which alleviated clinical symptoms rather than curing the disease. As a monogenic disease, gene therapy is considered a promising therapy. Multiple studies have transduced the *NF1-GRD* gene into different cell lines *via* various vectors and successfully reversed the cell phenotype (Hiatt et al., 2001; Thomas et al., 2006; Bodempudi et al., 2009; Bai et al., 2019).

Given the fact that CPT is a progressive disorder, early diagnosis and intervention are warranted to improve the prognosis of the disease. Owing to the complexity of clinical phenotypes, an extended genetic characterization of this multiple system disorder will be helpful in clinical diagnosis as well as treatment. Precise genetic counseling and gene diagnosis are recommended for CPT patients. If the proband in our study could get early gene detection and be provided with the plaster or cast support and specific nutrition support at an early age, the progression of bone deformities might be slowed down and his prognosis might be better. Therefore, based on the data in this study, we call for attention from pediatric orthopedists, other health care professionals, and patients’ caregivers and highly recommended screening for pathogenetic mutations.

## Data Availability

The datasets for this article are not publicly available due to concerns regarding participant/patient anonymity. Requests to access the datasets should be directed to the corresponding authors.
